# MicroRNA‐31a‐5p from aging BMSCs links bone formation and resorption in the aged bone marrow microenvironment

**DOI:** 10.1111/acel.12794

**Published:** 2018-06-12

**Authors:** Rongyao Xu, Xiang Shen, Yameng Si, Yu Fu, Weiwen Zhu, Tao Xiao, Zongyun Fu, Ping Zhang, Jie Cheng, Hongbing Jiang

**Affiliations:** ^1^ Jiangsu Key Laboratory of Oral Diseases Nanjing Medical University Nanjing China; ^2^ Department of Oral and Maxillofacial Surgery The Affiliated Stomatological Hospital of Nanjing Medical University Nanjing China

**Keywords:** aging, bone marrow stromal cells, exosomes, microRNA‐31a‐5p, osteoclasts, senescence‐associated heterochromatin foci

## Abstract

The alteration of age‐related molecules in the bone marrow microenvironment is one of the driving forces in osteoporosis. These molecules inhibit bone formation and promote bone resorption by regulating osteoblastic and osteoclastic activity, contributing to age‐related bone loss. Here, we observed that the level of microRNA‐31a‐5p (miR‐31a‐5p) was significantly increased in bone marrow stromal cells (BMSCs) from aged rats, and these BMSCs demonstrated increased adipogenesis and aging phenotypes as well as decreased osteogenesis and stemness. We used the gain‐of‐function and knockdown approach to delineate the roles of miR‐31a‐5p in osteogenic differentiation by assessing the decrease of special AT‐rich sequence‐binding protein 2 (SATB2) levels and the aging of BMSCs by regulating the decline of E2F2 and recruiting senescence‐associated heterochromatin foci (SAHF). Notably, expression of miR‐31a‐5p, which promotes osteoclastogenesis and bone resorption, was markedly higher in BMSCs‐derived exosomes from aged rats compared to those from young rats, and suppression of exosomal miR‐31a‐5p inhibited the differentiation and function of osteoclasts, as shown by elevated RhoA activity. Moreover, using antagomiR‐31a‐5p, we observed that, in the bone marrow microenvironment, inhibition of miR‐31a‐5p prevented bone loss and decreased the osteoclastic activity of aged rats. Collectively, our results reveal that miR‐31a‐5p acts as a key modulator in the age‐related bone marrow microenvironment by influencing osteoblastic and osteoclastic differentiation and that it may be a potential therapeutic target for age‐related osteoporosis.

## 1 INTRODUCTION

Aging in bone tissue is characterized by decreased bone formation and increased bone resorption, often leading to diseases such as osteoporosis (Guo, Chen, Guo, Jiang, & Lin, 2016; Wu et al., 2017). The balance between bone formation by osteoblasts and bone resorption by osteoclasts is important to maintain bone mass. Emerging evidence has demonstrated that the cross‐talk between monocyte‐macrophage‐osteoclasts and bone marrow stromal cells (BMSCs)‐osteoblasts plays a vital role in the pathology of osteoporosis (Cao et al., 2005; Yu, & Wang, 2016). With organismal aging, molecules of the senescence‐associated secretory phenotype (SASP) are secreted into the bone microenvironment by senescent cells (Ghosh, & Capell, 2016; Lim, Park, Shin, Kwon, & Kim, 2017). These molecules attenuate osteogenic differentiation as well as promote adipogenic differentiation and senescence of BMSCs, which is considered to be the primary cause of osteoporotic bone loss (Riminucci, Remoli, Robey, & Bianco, 2015; Tsai, & Li, 2017). Importantly, these SASP components could potentially transmit signals between BMSCs and osteoclasts, thereby regulating the status of bone remodeling. However, the molecular mechanisms underlying the functional impairment of BMSCs and communication between BMSCs and osteoclasts are not fully understood.

MicroRNAs (miRNAs), which function as the key post‐transcriptional repressors of gene expression in diverse cells, are a class of short noncoding RNAs (~22 nucleotides; Jing et al., 2015; Li, Cheng et al., 2015; Wang et al., 2013). They can inhibit target gene levels by binding to the 3′‐untranslated region (UTR) of specific mRNA targets and hence degrade the mRNA or suppress translation. miRNAs have been observed to participate in multiple biological processes, especially bone remodeling. Studies have demonstrated that several miRNAs, as a component of SASP, are released into the bone microenvironment and are involved in osteogenesis and osteoclastogenesis in the bone remodeling process (Chen et al., 2014; Wu et al., 2012; Yang et al., 2013). However, most miRNAs have been analysed intracellularly only, and few investigations have focused on the role of miRNAs in the extracellular environment. Furthermore, miRNAs that are secreted into the extracellular environment are easily degraded when they have no protection. Thus, the role of miRNA‐mediated cross‐talk between bone formation and resorption in age‐related bone loss remains elusive.

Exosomes are membrane‐bound phospholipid vesicles (40–150 nm in diameter) of endocytic origin (Chen, & Pfeifer, 2017; Guo, Tao et al., 2016; Im et al., 2014). Exosomes are intracellularly secreted by endocytic invagination of mammalian cells. These vesicles may contain proteins as well as mRNAs and miRNAs and have specific cargo (Greenhill, 2017; Mathivanan, Ji, & Simpson, 2010; Mead, & Tomarev, 2017). Upon secretion into the extracellular environment, exosomes have been demonstrated to carry their cargo to target cells, protecting the cargo from degradation during transportation (Sugatani, Hildreth, Toribio, Malluche, & Hruska, 2014; Xu, & Tahara, 2013). Therefore, the cross‐talk between BMSCs and osteoclasts may depend on the shuttling of exosomal miRNAs in the bone marrow microenvironment, which then modulates bone remodeling.

Our previous study demonstrated that microRNA‐31a‐5p (miR‐31a‐5p) in BMSCs regulates osteogenic differentiation in tooth eruption via the SATB2 pathway (special AT‐rich binding protein 2; Ge et al., 2015). Our previous results also demonstrated that SATB2 improves the stemness capacity and osteogenic differentiation of aged human BMSCs (Zhou et al., 2016). In addition, miR‐31a‐5p has been reported to directly target bone relevant markers, such as RUNX2, OSX, and DKK1 (Baglio, Devescovi, Granchi, & Baldini, 2013; Deng, Wu et al., 2013; Gao et al., 2011; Lv et al., 2017). Furthermore, it has been reported that regulation of miR‐31a‐5p expression could be used to modulate senescence‐related pathological conditions such as cancer, and the aging process (Capri et al., 2017; Cho, Dimri, & Dimri, 2015), which highlights us the important role of miR‐31a‐5p in cellular senescence. Moreover, according to a previous study, miR‐31a‐5p controls osteoclast formation and facilitates IL‐2 production in T cells by targeting RhoA (Fan et al., 2012; Mizoguchi, Murakami, Saito, Miyasaka, & Kohsaka, 2013). Thus, these findings raise the possibility that miR‐31a‐5p derived from BMSCs regulates both osteoblastogenesis and osteoclastogenesis.

Here, we identified a highly expressed miRNA, miR‐31a‐5p, in the BMSCs of aged rats and explored its role in modulating BMSCs and osteoclasts through secretion of exosomes. Our results demonstrated that BMSCs from aged rats exhibited decreased osteogenesis and increased aging phenotypes, which were associated with the upregulation of miR‐31a‐5p. Importantly, this is the first report that miR‐31a‐5p regulates cellular senescence mechanically by facilitating senescence‐associated heterochromatin foci (SAHF) formation. Moreover, blocking the functions of miR‐31a‐5p enhanced osteoblastogenesis and reduced osteoclastogenesis both in vitro and in vivo. Thus, our study provides a potential therapeutic approach for age‐related osteoporosis.

## 2 RESULTS

### 2.1 Aged‐related increase of miR‐31a‐5p in BMSCs

**Figure 1:**
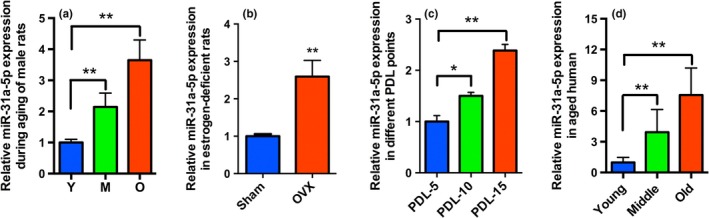
Aged‐associated increase of miR‐31a‐5p in BMSCs. (a) miR‐31a‐5p expression was increased in BMSCs from aged rats compared to that in the young (Y) and elderly rats (O) as analysed by qRT‐PCR,* n* = 5. (b) miR‐31a‐5p was detected at high levels in BMSCs from ovariectomized (OVX) rats versus BMSCs from sham rats, *n* = 5. (c) Significantly increased miR‐31a‐5p was observed in BMSCs at PDL‐15 versus BMSCs from early passages, *n* = 5. (d) Human BMSCs demonstrated that miR‐31a‐5p expression was increased with aging, *n* = 10. *p <* *0.05, **p <* *0.01. Data are presented as the mean ± *SD*

To investigate the expression of miR‐31a‐5p during aging, four aging‐associated models were established for analysis by qRT‐PCR. We performed micro‐CT to observe the bone loss of aged male rats and ovariectomized (OVX) rats (Supporting information Figure S1A and B). BMSCs from elderly rats (O) demonstrated an increased expression level of miR‐31a‐5p compared to the levels in middle‐aged rats (M) and their young counterparts (Y; Figure 1a). To study the relationship of estrogen‐deficient bone loss with miR‐31a‐5p expression, miR‐31a‐5p levels in BMSCs from OVX rats were assessed and were observed to be elevated compared to that in the controls (Figure 1b). To examine whether miR‐31a‐5p expression was altered during replicative senescence, SA‐β‐gal staining and expression of miR‐31a‐5p in BMSCs at PDL‐15 were analysed and were observed to be significantly increased compared to that in BMSCs at PDL‐10 and 5 (Supporting information Figure S1C and Figure 1c). Expression of miR‐31a‐5p in human BMSCs was also significantly increased with aging (Figure 1d). An inverse correlation between bone mineral density (BMD) and age was found (*R* ^2^ = 0.8117; *y* = −0.9547*x* + 157.17) as shown in Supporting information Figure S1D, which indirectly indicates that expression of miR‐31a‐5p is negatively correlated with BMD in human alveolar bone. The capacities of osteoblastogenesis, adipogenesis, and aging phenotype of young and aged human BMSCs were also examined (Supporting information Figure S1E‐G). Among these aging models, we focused our analysis on aged rats, which represent a suitable model to mimic human osteoporosis with organismal aging, for further investigation. Our results suggested that expression of miR‐31a‐5p is significantly elevated with increasing age. These results revealed the potential role of miR‐31a‐5p from BMSCs in age‐related bone loss.

### 2.2 Aged‐related properties of BMSCs

**Figure 2:**
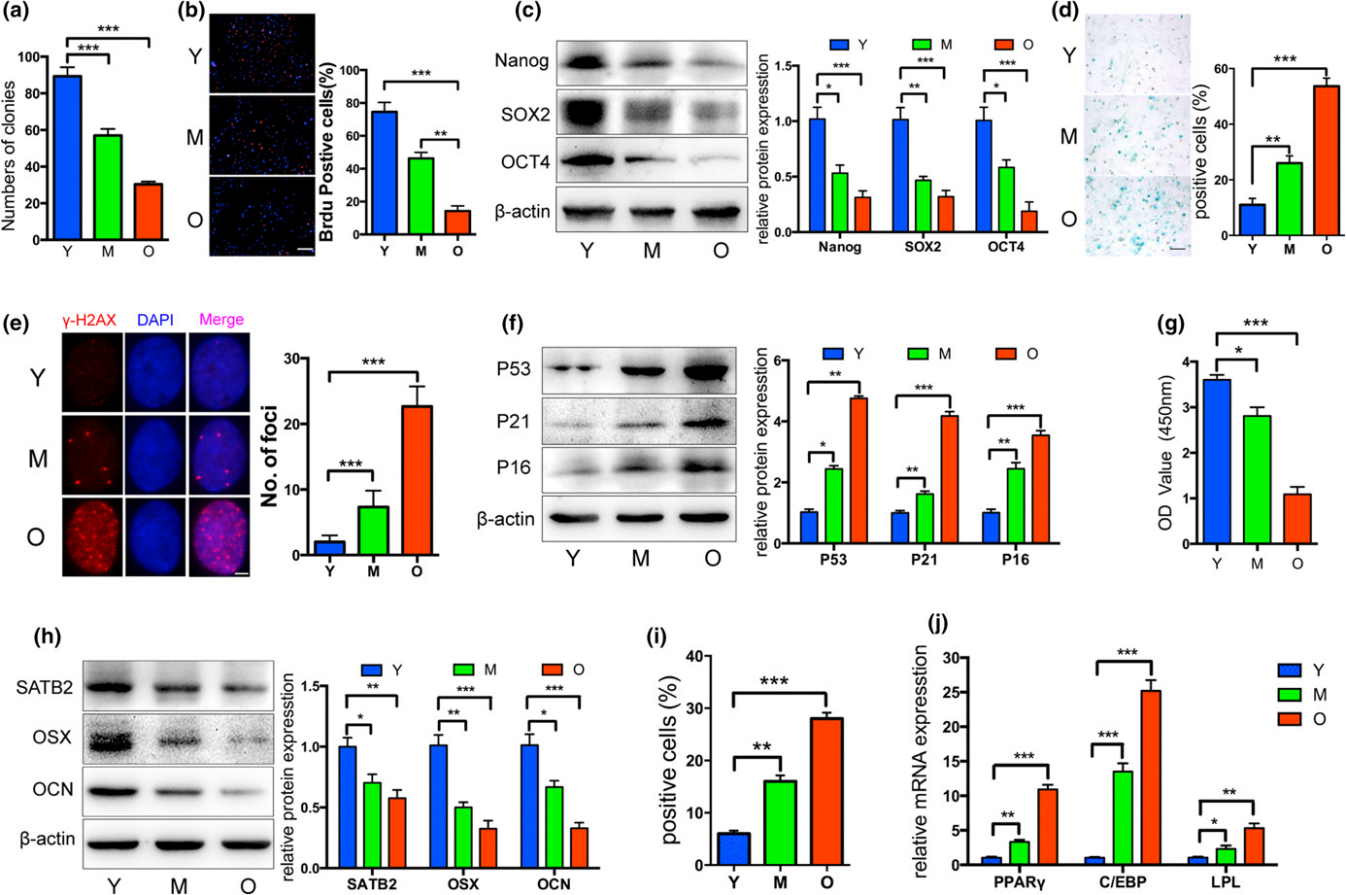
Properties of BMSCs from aged rats. (a) Decreased colony forming units were observed in BMSCs from the O group compared to those from the Y and M (young and middle‐aged rats) groups. (b) The ability of proliferation was significantly reduced in elderly BMSCs, as shown by the BrdU assay. (c) Western blot showed decreased Nanog, SOX2, and OCT4 protein levels in aged BMSCs. (d and e) SA‐β‐gal staining and γH2AX foci formation exhibited more senescent cells in the O group. (f) Senescence‐related proteins P53, P21, and P16 were upregulated in the aged BMSCs. (g) Reduced osteogenic differentiation of aged BMSCs was observed by Alizarin red staining. (h) Western blot revealed that the special AT‐rich sequence‐binding protein 2 (SATB2) and osteogenic markers osterix (OSX) and osteocalcin (OCN) were significantly decreased in BMSCs from the O group. (i and j) Increased adipogenic ability of aged BMSCs was detected by oil red O staining, and higher mRNA levels of adipogenic markers were observed in the O group relative to those in the Y and M groups. *p <* *0.05, **p <* *0.01, ***p <* *0.001. Scale bars: 100 μm (b); 100 μm (d); 4 μm (e). Data are presented as the mean ± *SD*,* n* = 3

To understand the properties of aged BMSCs, we examined stemness and aging in vitro. The colony‐forming rates (Figure 2a and Supporting information Figure S2A) and proliferation (Figure 2b) of aged BMSCs were significantly reduced compared to those from the Y and M groups. Consistent with these results, expression of the stemness factors Nanog, SOX2, and OCT4 were remarkably decreased with increasing age (Figure 2c). As anticipated, a greater number of SA‐β‐gal‐positive BMSCs at PDL‐5 was observed in the O group than in the Y and M groups (Figure 2d). The results of the assay for γH2AX foci formation—another marker of cell senescence—exhibited a trend similar to that of SA‐β‐gal staining (Figure 2e). In line with these findings, the senescence‐related factors P53, P21 and P16 were markedly upregulated in the O group compared to the levels in the Y and M groups (Figure 2f). Altogether, these data revealed that a decline of stemness and a build‐up of the aging phenotype occurred in the O group, leading to a significant functional compromise in BMSCs.

BMSCs derived from elderly rats exhibited decreased osteogenesis and increased adipogenesis. Following 14 days of osteogenic induction of BMSCs at PDL‐3, mineral deposits were evidently reduced in the O group and were quantified after controlling for the cell numbers of each group (Figure 2g and Supporting information Figure S2B). In parallel with the SATB2 decline, the levels of osteogenic marker proteins, such as osterix (OSX) and osteocalcin (OCN), were also reduced in the M and O groups (Figure 2h), which was in agreement with the results of mRNA expression of Osx and Ocn compared to the expression levels in the Y group after 7 days of osteogenic induction (Supporting information Figure S2C). After 14 days of adipogenic induction, elderly rat‐derived BMSCs at PDL‐3 exhibited an enhanced adipogenic potential, as indicated by the increased number of lipid droplets as well as elevated expression of adipogenic markers relative to the levels in the Y and M groups (Figure 2i,j and Supporting information Figure S2D). These data suggested that BMSCs demonstrate reduced osteogenic potential with increasing age and a more likely differentiation toward adipogenesis, which is a predominantly pathological process involved in osteoporosis.

### 2.3 SATB2 is a functional target of miR‐31a‐5p involving in BMSCs osteogenic differentiation

**Figure 3:**
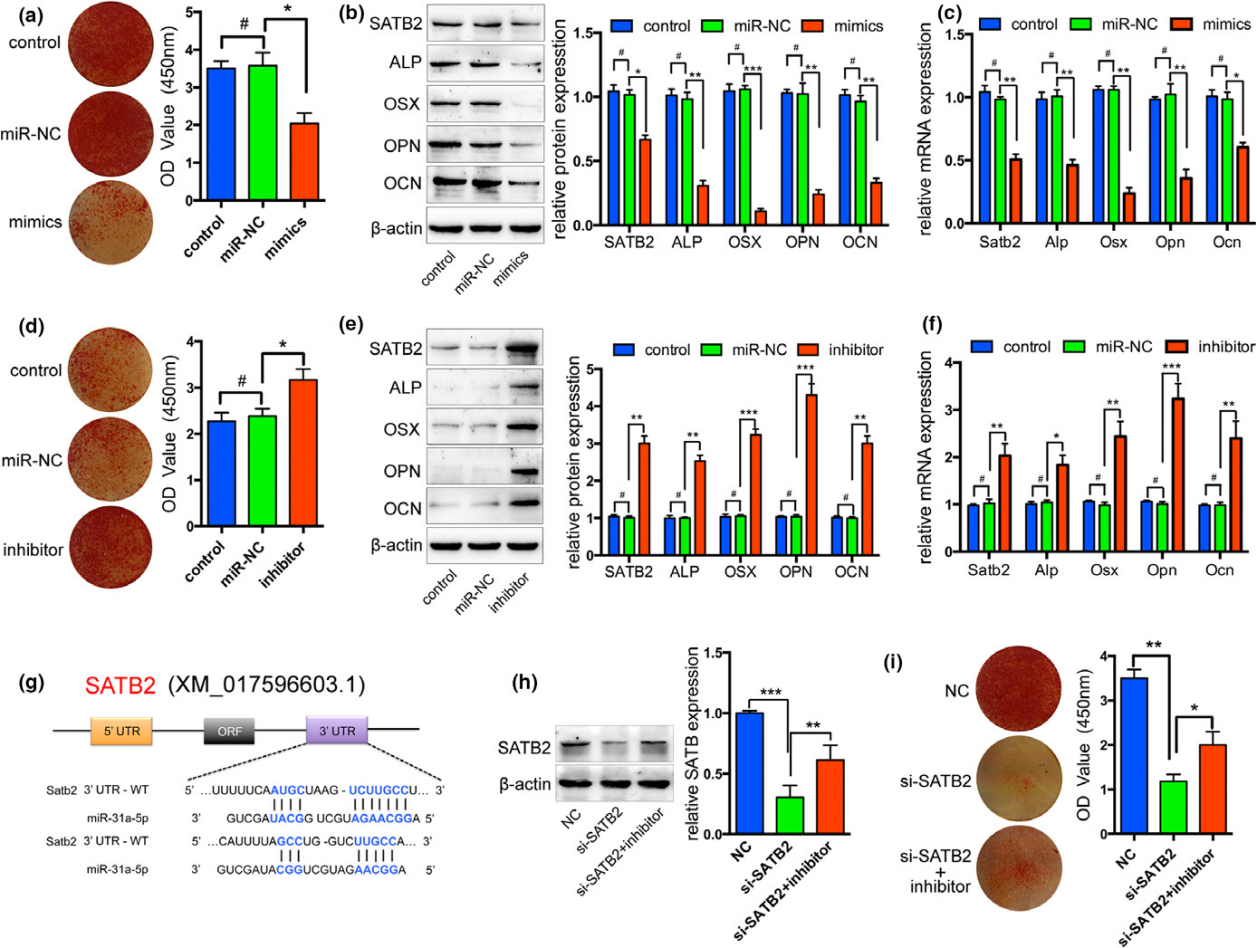
miR‐31a‐5p regulates the downstream expression of genes in the SATB2 pathway. (a) miR‐31a‐5p mimics remarkably decreased the number of mineral deposits of BMSCs. (b and c) In transfected cells, Western blot and qRT‐PCR showed that the expression of SATB2 and the osteogenic markers, OSX and OCN, were also reduced by miR‐31a‐5p mimics. (d) Inhibition of miR‐31a‐5p dramatically increased mineral deposits and (e and f) enhanced osteogenic markers. (g) SATB2 was predicted as the target of miR‐31a‐5p by TargetScan. (h) Western blot showed the SATB2 expression and (i) Alizarin red staining reveals that inhibition of miR‐31a‐5p rejuvenated the compromised osteogenic capacity of si‐SATB2 treated BMSCs. ^*#*^ *p *>* *0.05, *p <* *0.05, **p <* *0.01, ***p <* *0.001. Data are presented as the mean ± *SD*,* n* = 3

Since decreased osteogenesis and increased aging phenotype of BMSCs were accompanied by enhanced expression of miR‐31a‐5p, we investigated whether miR‐31a‐5p could regulate osteogenesis and aging. After 14 days of osteogenic induction, fewer mineral deposits were found in young rats derived BMSCs transfected with miR‐31a‐5p mimics (Figure 3a). In the transfected cells, osteogenic markers, such as alkaline phosphatase (ALP), OSX, osteopontin (OPN), and OCN, displayed a similar pattern (Figure 3b,c). Furthermore, miR‐31a‐5p inhibition in elderly rat‐derived BMSCs produced more mineral deposits and increased expression of osteogenic markers compared to their expression in the control and miR‐NC group (Figure 3d–f). The reduced protein levels of SATB2 suggested that expression of miR‐31a‐5p was negatively correlated with SATB2 levels. We used TargetScan to predict the target of miR‐31a‐5p and found that miR‐31a‐5p could bind to the 3′‐untranslated region (3′UTR) of SATB2 (Figure 3g), a pluripotency transcription factor, which has been previously demonstrated (Deng, Wu et al., 2013; Ge et al., 2015). To further demonstrate whether miR‐31a‐5p regulated osteogenesis via SATB2, the osteogenic capacity of si‐SATB2 treated BMSCs was rejuvenated by miR‐31a‐5p inhibition (Figure 3h,i). Thus, our results revealed that inhibition of miR‐31a‐5p contributes to increased osteogenic differentiation of BMSCs through the SATB2 pathway.

### 2.4 miR‐31a‐5p directly bound to the 3′UTR of E2F2 and promoted SAHF formation in aged cells

**Figure 4:**
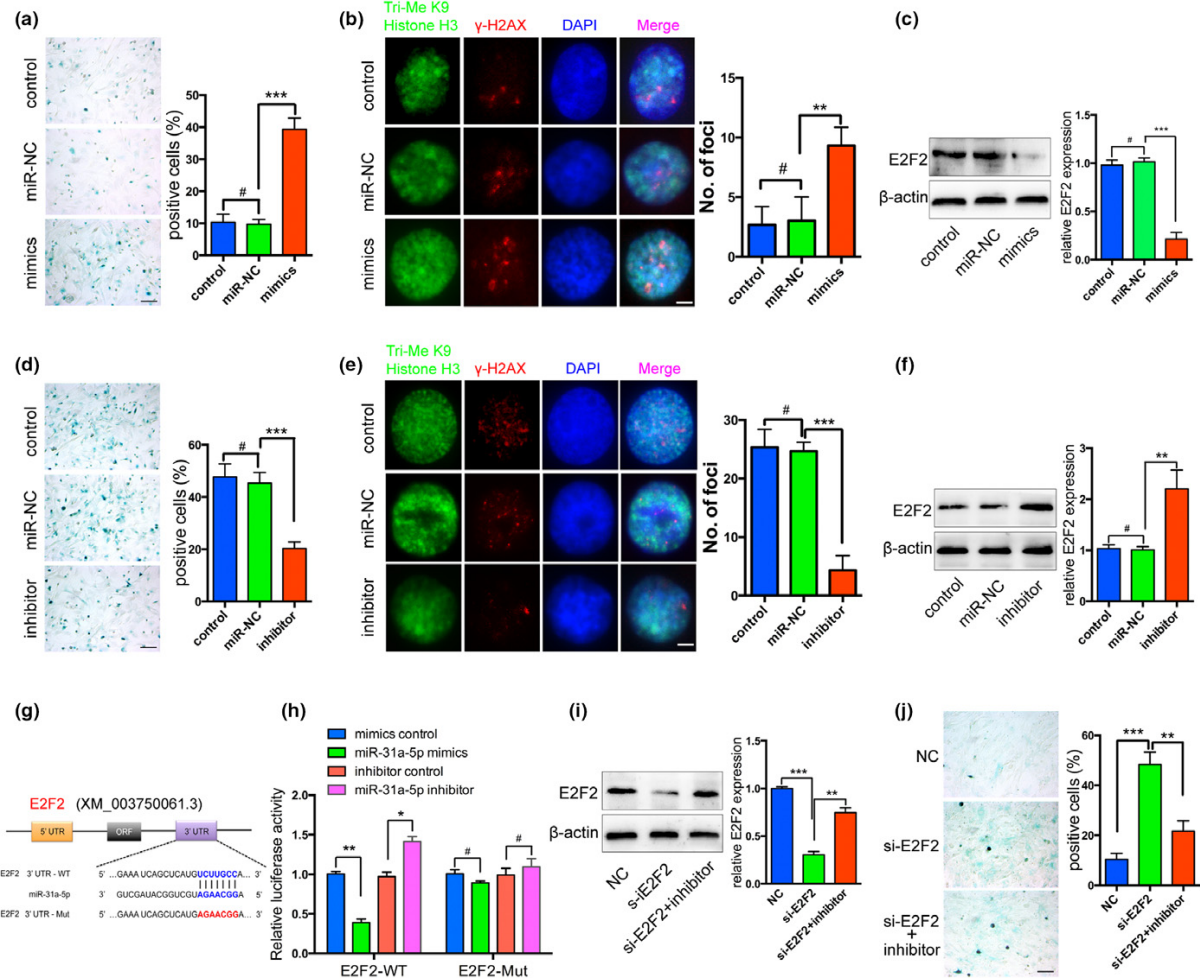
miR‐31a‐5p inhibition rejuvenates the aging phenotype of BMSCs via the E2F2 pathway. (a and d) Representative images of SA‐β‐gal staining in BMSCs transfected with miR‐31a‐5p mimics, miR‐31a‐5p inhibitor, or their controls were presented. (b and e) Overexpression or inhibition of miR‐31a‐5p in BMSCs was confirmed by γH2AX and H3K9me3 immunostaining. (c and f) The protein expression levels of E2F2 were reduced by the miR‐31a‐5p mimics and increased by miR‐31a‐5p suppression in BMSCs. (g) The potential binding sites for miR‐31a‐5p on the 3′UTR of E2F2. (h) 293T cells were transfected with a luciferase reporter vector containing either the WT or mutant plasmid of the 3′UTR of E2F2. (i) E2F2 protein was measured by Western blot and (j) miR‐31a‐5p inhibition significantly reduced SA‐β‐gal‐positive cells in si‐E2F2 treated BMSCs. ^*#*^ *p *>* *0.05, *p <* *0.05, **p <* *0.01, ***p <* *0.001. Scale bars: 100 μm (a, d and j); 4 μm (b and e). Data are presented as the mean ± *SD*,* n* = 3

Next, we explored the role of miR‐31a‐5p in cellular aging. miR‐31a‐5p mimics in BMSCs caused a significant increase in the number of SA‐β‐gal‐positive cells (Figure 4a) and inhibition of miR‐31a‐5p dramatically attenuated positive staining cells compared to those observed in the control group (Figure 4d). Remarkably, γH2AX foci were enriched in miR‐31a‐5p overexpressing cells and declined in miR‐31a‐5p inhibition cells (Figure 4b,e). Since γH2AX foci are reminiscent of SAHF, we observed that histone H3 tri‐methylated Lys9 (H3K9me3), which are heterochromatic epigenetic marks, were also assembled in the nuclei consistent with γH2AX enrichment (Figure 4b,e). We therefore hypothesized that miR‐31a‐5p association with SAHF loci allows for the induction of cellular aging. The databases of Targetscan (http://www.targetscan.org), miRanda (http://www.microrna.org), and miRDB (http://mirdb.org) were used for bioinformatics analysis to predict the target gene of miR‐31a‐5p. Subsequently, E2F2, a member of the E2Fs family, was screened out because inhibiting the transcriptional activation function of E2Fs led to cellular senescence and SAHF assembly. Western blot revealed that the expression of miR‐31a‐5p was negatively correlated with the E2F2 levels (Figure 4c,f). To ascertain whether miR‐31a‐5p directly binds to the 3′UTR of E2F2 and causes translational inhibition, we employed dual‐luciferase reporter assays in 293T cells. The miR‐31a‐5p mimics significantly decreased the luciferase activity of the wild‐type (WT) reporter, but did not affect the luciferase activity of the mutant reporter. The effect of the miR‐31a‐5p inhibitor on either WT or mutant reporter luciferase activity further suggested that miR‐31a‐5p directly binds to the 3′UTR of E2F2 (Figure 4g,h). To further confirm whether miR‐31a‐5p promoted the aging of BMSCs by E2F2, the SA‐β‐gal‐positive cells treated with si‐E2F2 were found to be significantly reduced by miR‐31a‐5p inhibition (Figure 4i,j). These results supported the hypothesis that miR‐31a‐5p induces cellular aging by blocking E2F2 activity and leading to SAHF assembly.

### 2.5 Exosomal miR‐31a‐5p from BMSCs increases osteoclastic numbers and function

**Figure 5:**
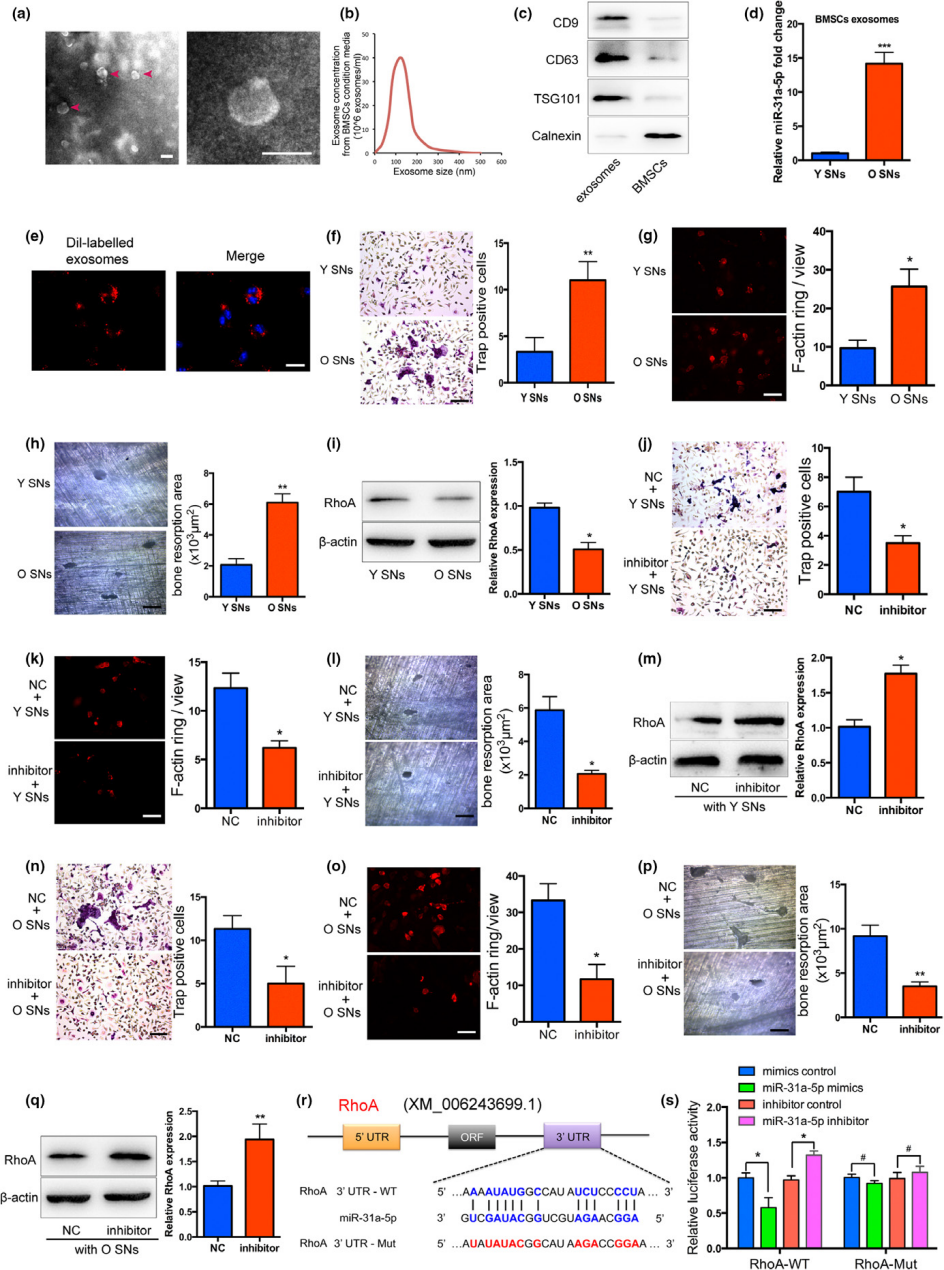
Exosomal miR‐31a‐5p derived from BMSCs inhibits osteoclastogenesis and bone resorption. (a) Exosomes were visualized on a transmission electron microscope (TEM) and the red arrows indicate exosomes. (b) NTA analysis determined the size distribution of the isolated microvesicles. (c) Western blot of CD9, CD63, TSG101, and Calnexin was performed on the exosomes and BMSCs. (d) Exosomal miR‐31a‐5p was significantly increased in the supernatant from aged BMSCs compared to that in the Y group, as detected by qRT‐PCR. (e) DiI‐labeled exosomes from BMSCs incorporated into osteoclasts were visualized by fluorescent microscopy analysis. (f–h) Exosomes derived from aged BMSCs promoted osteoclastic differentiation, as detected by Trap and F‐actin ring staining, and led to increased bone resorption area on the dentin disk treated with the supernatant from the O group. (i) Osteoclasts treated with the O group supernatant exhibited decreased RhoA protein levels. Inhibition of exosomal miR‐31a‐5p in the (j–l) Y group supernatant or (n–p) O group supernatant resulted in decreased osteoclasts and reduced bone resorption area on the dentin disk relative to the levels in the control group. (m,q) RhoA activity was significantly increased in the osteoclasts treated with supernatant from young or elderly rats containing the miR‐31a‐5p inhibitor. (r) Identification of miR‐31a‐5p binding sites on the 3′UTR of RhoA by bioinformatics analysis. (s) Dual‐luciferase reporter assays were performed to confirm the result that miR‐31a‐5p directly targeted the 3′UTR of RhoA. ^*#*^ *p *>* *0.05, *p <* *0.05, **p <* *0.01, ***p <* *0.001. Scale bars: 100 nm (a); 50 μm (e); 100 μm (f–h); 100 μm (j–l); 100 μm (n–p). SNs: supernatants. Data are presented as the mean ± *SD*,* n* = 3

Recently, exosomes were shown to serve as vehicles for microRNAs, protecting them from degradation and transporting them to target cells. The morphology of purified microvesicles was observed by TEM to identify exosomes (Figure 5a). The size distribution of the isolated microvesicles was subjected to nanoparticle tracking analysis (NTA; Figure 5b). CD9, CD63 and TSG101 (exosomal markers) were highly expressed as detected by Western blot in the exosome pellet, while Calnexin (a cytosolic marker) was barely detectable in exosomes (Figure 5c). miR‐31a‐5p was detected at high levels in the exosomes contained in the supernatant derived from elderly BMSCs (Figure 5d). Supernatant containing DiI‐labeled exosomes from BMSCs were co‐cultured with osteoclasts to demonstrate that exosomes were incorporated into osteoclasts (Figure 5e).

To analyse the function of exosomal miR‐31a‐5p from BMSCs in osteoclastogenesis, bone marrow cells treated with supernatant from young or elderly rat samples were induced towards osteoclastogenesis and cultured on a dentin disk. The data demonstrated that more multinucleated osteoclasts were observed after treatment with the supernatant from elderly rats compared to those observed in the counterpart groups. In line with this finding, increased activity of bone resorption was found on the dentin disk treated with supernatant from elderly rats (Figure 5f–h). After being treated with the negative control or miR‐31a‐5p inhibitors in the young rat‐derived supernatant, exosomes were cocultured with bone marrow cells. Following a period of osteoclastic induction, the number of multinucleated osteoclasts and the area of the resorption pits on the dentin surfaces were significantly decreased in the cocultured condition after treatment with miR‐31a‐5p inhibitors in contrast to the control group (Figure 5j–l). Exosomes treated with miR‐31a‐5p inhibition in elderly rat‐derived supernatant also decreased osteoclast numbers, and repressed osteoclast function in the cocultured condition (Figure 5n–p). By bioinformatics analysis, RhoA, which regulates the cytoskeleton organization of osteoclasts, was predicted as the putative target gene of miR‐31a‐5p (Figure 5r). Our results showed that RhoA was significantly decreased in osteoclasts treated with supernatant from elderly rats (Figure 5i) and the impaired function of osteoclasts was accompanied by enhanced expression of RhoA (Figure 5m,q). To confirm whether miR‐31a‐5p directly binds to the 3′UTR of RhoA, we performed dual‐luciferase reporter assays in 293T cells. Our results verified that miR‐31a‐5p directly binds to the 3′UTR of RhoA and causes translational inhibition (Figure 5s). Thus, these results indicated that inhibition of miR‐31a‐5p in exosomes can reduce osteoclastogenesis and bone resorption by elevating RhoA expression at the translational level.

### 2.6 AntagomiR‐31a‐5p delivery prevents bone loss in aged rats

**Figure 6:**
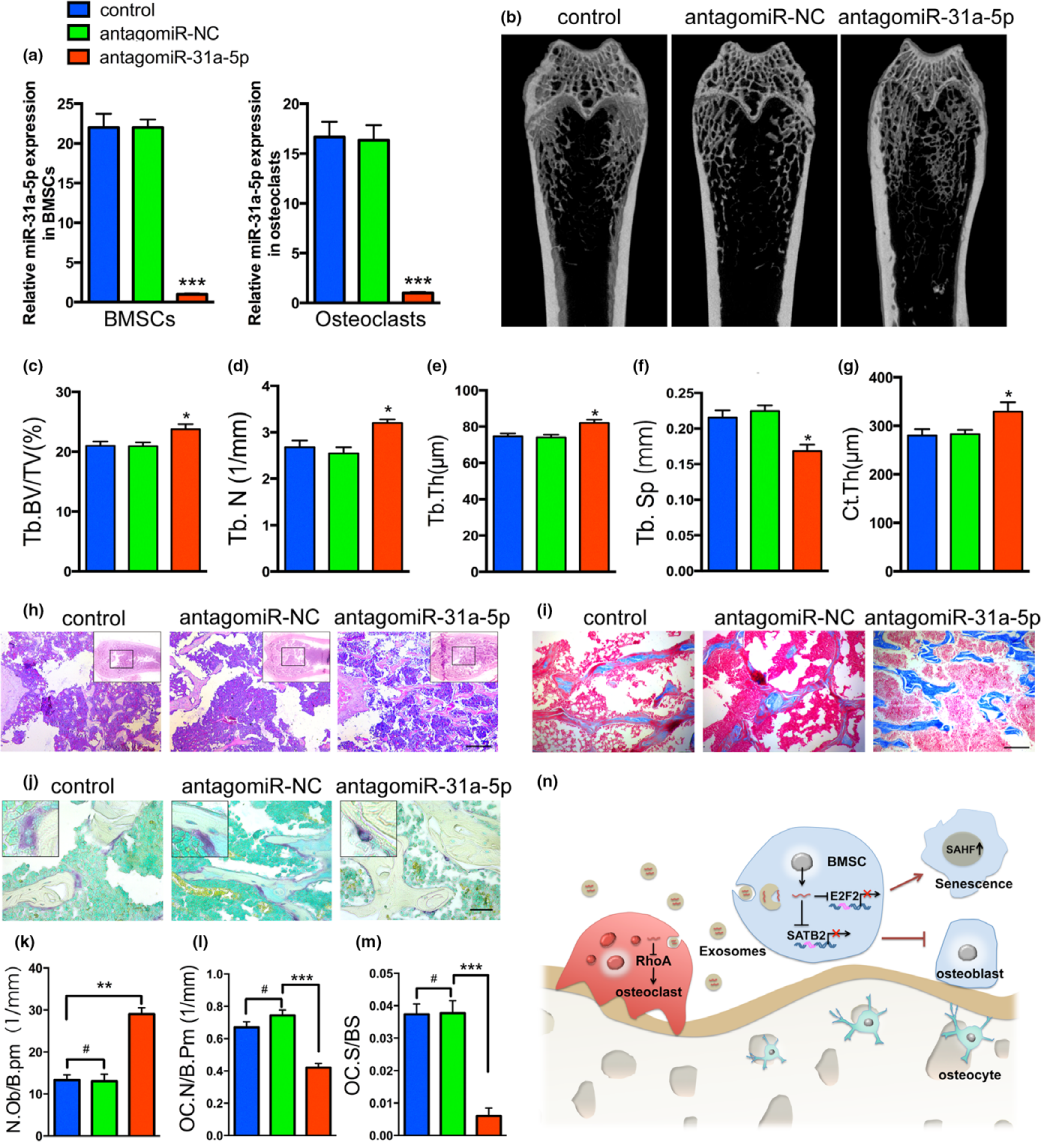
Injection of antagomiR‐31a‐5p into the bone marrow cavity stimulates bone formation and reduces osteoclastogenesis in aged rats. (a) qRT‐PCR analysis of levels of miR‐31a‐5p expression in BMSCs and osteoclasts of rats with antagomiR‐31a‐5p. (b) Micro‐CT images and quantitative CT analysis (c–g) were performed in the distal femur from 15‐month‐old rats treated with control, antagomiR‐NC and antagomiR‐31a‐5p. (h and i) Representative images of HE and Masson's trichrome staining exhibited that bone structure and (k) osteoblasts were significantly increased in aged rats injected with antagomiR‐31a‐5p. (j) Trap staining of distal femur in the three groups and (l) quantification of OC.N/B.Pm (osteoclast number per bone perimeter) and (m) OC.N/BS (osteoclast number per bone surface) indicated that osteoclast numbers were significantly decreased on the bone surface with antagomiR‐31a‐5p treatment compared to those with antagomiR‐NC and control treatment. (n) A schematic working model for miR‐31a‐5p function in the regulation of BMSCs and osteoclast. ^*#*^ *p *>* *0.05, *p <* *0.05, **p < 0.01, ***p <* *0.001. Scale bars: 200 μm (h); 100 μm (i); 50 μm (j). Data are presented as the mean ± *SD*,* n* = 3

Before injection of antagomiR‐31a‐5p into the bone marrow, the qRT‐PCR results confirmed that BMSCs treated with antagomiR‐31a‐5p led to a significant decrease in the level of miR‐31a‐5p, as well as in osteoclasts (Figure 6a). To determine the therapeutic function of miR‐31a‐5p inhibition in age‐related osteoporosis, 15‐month‐old rats were administered antagomiR‐31a‐5p or mutant antagomiR‐31a‐5p in the femoral bone marrow cavity. Three months after administration, bone marrow injected with antagomiR‐31a‐5p demonstrated significantly increased trabecular bone volume, thickness and trabecular numbers as well as decreased trabecular separation compared to the levels in the control group (Figure 6b–f). The cortical bone thickness was also higher in the femurs of antagomiR‐31a‐5p treated rats compared with the control‐treated rats (Figure 6g). Notably, antagomiR‐31a‐5p enhanced trabecular bone structure (Figure 6h,i) and increased the number of osterix‐positive preosteoblasts (Supporting information Figure S3) and osteoblasts on trabecular bone surfaces in bone marrow (Figure 6k). Moreover, rats treated with antagomiR‐31a‐5p showed significantly lower osteoclast numbers compared to those in the control rats (Figure 6j,l,m). These results suggested that bone tissue treated with antagomiR‐31a‐5p prevents bone loss and decreases osteoclastic differentiation in aged rats.

## 3 DISCUSSION

The present study demonstrated that miR‐31a‐5p derived from aged BMSCs reduced osteoblastogenesis and promoted osteoclastogenesis in aging bone tissue, leading to osteoporotic bone loss (Figure 6n). Briefly, miR‐31a‐5p was significantly increased with the aging of BMSCs and was secreted into the extracellular microenvironment via exosomes. Then, miR‐31a‐5p, using exosomes as vehicles, not only regulated BMSCs function in osteogenesis and cellular aging by the SATB2 and E2F2 pathways, respectively, but also positively regulated osteoclastic differentiation by the RhoA pathway. Furthermore, application of antagomiR‐31a‐5p into bone marrow reduced age‐associated bone loss, which suggests it may be a potentially biological therapy for age‐related osteoporosis.

It has been reported that miR‐31a‐5p negatively regulates skeletogenesis and osteogenesis (Deng, Wu et al., 2013; Jin et al., 2016; Stepicheva, & Song, 2015; Weilner et al., 2016). Previous studies also demonstrated that decreased stemness and increased senescence are the primary causes of declines in osteogenic differentiation of BMSCs (Fehrer, & Lepperdinger, 2005; Zhou et al., 2016). In this study, we observed that the stemness and osteogenic differentiation of BMSCs were significantly reduced with increasing age, while the aging phenotype and adipogenic differentiation of aged BMSCs were markedly increased. Importantly, these phenotypic changes in BMSCs with aging were accompanied by progressively enhanced miR‐31a‐5p expression. The increased level of miR‐31a‐5p was further confirmed by the estrogen‐deficient aging model, replicative aging model and human aging model. SATB2 is a target gene of miR‐31a‐5p, which has been proven by some prior studies (Aprelikova et al., 2010; Deng, Weng et al., 2013; Deng, Zhou et al., 2013). Our previous investigation has demonstrated that SATB2, as a transcriptional regulator, functions broadly (Dong et al., 2015) and is involved in osteogenic differentiation in age‐related BMSCs (Strickland, Wasserman, Giassi, Djordjevic, & Parra‐Herran, 2016; Zhou et al., 2016). Our results from both knockdown and gain‐of‐function approaches showed that miR‐31a‐5p inhibited osteogenic differentiation by negatively regulating SATB2 expression. Therefore, based on this evidence, we reasoned that the miR‐31a‐5p‐SATB2 axis plays a crucial role in osteogenic differentiation of BMSCs in osteoporotic bone loss during aging.

An important aspect of our findings concerns the SAHF. Cellular senescence is a permanent proliferation‐arrest induced by telomere shortening, oncogene activation or aging (Chandra et al., 2015; van Deursen, 2014). This process usually leads to SAHF formation, which consists of some transcriptional silencing markers such as heterochromatin protein 1 (HP1) and H3K9Me3 (Maeng, Kwon, Kim, & Kwon, 2015). When DNA is damaged either by laser or aging, E2Fs (E2F1‐3) target genes, such as CyclinA/E, PCNA, and MCM4, are compacted within the SAHF to prevent cell proliferation, and this process is generally a consequence of retinoblastoma (Rb)‐mediated repression of E2Fs activity (Vernier et al., 2011; Zhao et al., 2015). Therefore, inhibition of E2Fs activity induces cellular senescence by recruiting SAHF foci in the nucleus. Furthermore, most γH2AX, which facilitate DNA double‐strand breaks (DSB) repair within the heterochromatin region, are spatially juxtaposed around with SAHF (Goodarzi et al., 2008). In our study, miR‐31a‐5p mimics gave rise to increased γH2AX assembly. Thus, the spatial relationship between SAHF and γH2AX made us ask whether miR‐31a‐5p is able to regulate SAHF formation during this mechanism. According to a previous study (Li, Luo, Liu, Lv, & Xi, 2015) and bioinformatics analysis, we found E2F2, one of the E2Fs family, is the target gene of miR‐31a‐5p. Our results also confirmed that miR‐31a‐5p bound to the 3′UTR of E2F2 and resulted in the induction of SAHF. Thus, these data suggest that miR‐31a‐5p contributes to cellular aging by promoting SAHF formation and compromising the BMSCs function leading to reduced osteoblastogenesis.

In the past few years, miRNAs have rapidly emerged as important regulators of gene expression by influencing translation and mRNA stability. They can induce changes in multiple cellular processes, including cell proliferation, cell differentiation, and cell senescence (Davis et al., 2017; Jing et al., 2015). However, their instability in the extracellular environment limits their application. Exosomes, as specialized membranous vesicles, protect miRNA from degradation and transfer them between cells to modulate cell‐cell communication. Our study demonstrated that exosomal miR‐31a‐5p derived from aged BMSCs was secreted into the extracellular environment and was significantly increased compared to the levels in young BMSCs. The number and function of osteoclasts were significantly increased when cells were cocultured with the supernatant from elderly rat samples. However, miR‐31a‐5p reduction in the supernatant not only led to decreased osteoclastogenesis and bone resorption but also elevated expression of RhoA. Although studies have demonstrated that RhoA activity, controlling cytoskeleton organization, is crucial for osteoclast maturation and bone resorption (Chellaiah et al., 2000), it has also been reported that inhibition of RhoA maintained the podosomal and sealing zone stability, which is possibly correlated with increased microtubule acetylation and stabilization in osteoclasts (Destaing et al., 2005; Granot‐Attas, Luxenburg, Finkelshtein, & Elson, 2009; Ory, Brazier, Pawlak, & Blangy, 2008; Wu et al., 2017). In our study, increased RhoA expression through miR‐31a‐5p inhibition reduced osteoclastogenesis, while decreased RhoA expression favored osteoclastogenesis. Hence, our findings suggest that exosomal miR‐31a‐5p derived from BMSCs promotes the differentiation of osteoclasts by targeting the 3′UTR of RhoA and repressing its activity. Collectively, our results indicate that BMSC‐derived miR‐31a‐5p, which is packaged by exosomes, not only affects osteogenic differentiation but also osteoclastic differentiation through the shuttling of exosomes between cells. This double‐regulation indicates that miR‐31a‐5p functions as an essential regulator both in bone formation and bone resorption during aging.

Emerging evidence supports the hypothesis that accumulation of senescent cells or alteration of some age‐related molecules (e.g., miRNA, RNA, lipids, and proteins) packaged by exosomes in the bone microenvironment is the primary cause of osteoporosis (Farr et al., 2017; Gibon, Lu, & Goodman, 2016; Lim et al., 2017; Sui, Hu, Zheng, & Jin, 2016; Williams, Smith, Kumar, Vijayan, & Reddy, 2017). Targeting these exosomes, which act as important communicators between cells, may emerge as a new therapeutic approach for osteoporosis (Liu et al., 2017; Long et al., 2017; Nakano et al., 2016). In this study, we used antagomiR‐31a‐5p, which was injected into the bone marrow microenvironment, to decrease miR‐31a‐5p expression in the bone marrow microenvironment of osteoporotic rats. Our results suggested that antagomiR‐31a‐5p enhanced trabecular bone structure and repressed osteoclast numbers in aged bone tissue. Thus, we believe that application of antagomiR‐31a‐5p into the bone marrow microenvironment may be a viable therapeutic approach for osteoporosis.

In summary, our findings support the hypothesis that exosomal miR‐31a‐5p functions as an important regulator of both osteoblastogenesis and osteoclastogenesis. Moreover, application of antagomiR‐31a‐5p to the bone marrow microenvironment may provide a potential therapeutic strategy for age‐related bone loss.

## 4 EXPERIMENTAL PROCEDURES

### 4.1 Experimental animals

All experiments were performed with the approval of the Ethics Committee of the School of Stomatology of Nanjing Medical University. All procedures were carried out according to the guidelines of the Animal Care Committee of Nanjing Medical University. Three‐month‐old female Sprague‐Dawley rats were randomly divided into two groups. Each group underwent either sham surgery or bilateral ovariectomy via the dorsal approach (Xu et al., 2016). Three months after surgery, BMSCs in the femur and tibia were harvested from both sham and OVX rats (Fu et al., 2014). Fifteen‐month‐old male Sprague‐Dawley rats were randomly divided into three groups. Two groups received either antagomiR‐31a‐5p (1 μM) or mutant antagomiR‐31a‐5p (1 μM, antagomiR‐NC) twice per month for 3 months at a dose of 20 μl by periosteal injection into the marrow cavity of the femur. The other group of rats received a comparable volume of PBS (control; Li, Cheng et al., 2015). AntagomiR‐31a‐5p (CAGCUAUGCCAGCAUCUUGCCU) and mutant antagomiR‐31a‐5p (CAGUACUUUUGUGUAGUACAA) were synthesized by RiboBio Co. miRNA antagomir, which is specially labeled and chemically modified single‐stranded small RNAs, has high stability and can maintain activity for at least 2 weeks in bone marrow environments and regulate the target genes by directly penetrating the cell membrane without the need for transfection reagents. Rats were killed by CO_2_ inhalation in the third month, and femur samples were harvested and processed for further analysis (*n* = 3 per group).

### 4.2 In vitro culture and differentiation of BMSCs

BMSCs from young rats (3 months old), middle‐aged rats (12 months old) and aged rats (18 months old) were harvested from the femur and tibia as previously described in an earlier study (Fu et al., 2014). Briefly, bone marrow was flushed with α‐MEM by an 18‐gauge sterile needle inserted into the medullary cavity. For replicative senescence analysis, BMSCs from middle‐aged male rats were repeatedly divided by two and subcultured to a population doubling level (PDL)‐5, 10, and 15. BMSCs in the alveolar bone of the mandible from female humans were harvested from volunteer donors who underwent dental implantation or impacted tooth extraction at our hospital. Informed consent was obtained before volunteers were involved in this study. Inclusion criteria: (a) no jaw bone tumor or tumor‐like lesion; (b) no jaw bone fracture; (c) no intake of drugs affecting bone metabolism (corticosteroids, antiepileptic drugs, antituberculosis drugs, antacids containing aluminum, heparin); (d) no systemic disease that affected bone metabolism (hyperparathyroidism, hypoparathyroidism, osteochondrosis, renal osteodystrophy, deformans osteitis, osteogenesis imperfecta, diabetes). Thirty volunteers were divided into the young (Young, 19–27 years old), middle‐aged (Middle, 32–47 years old), and old group (Old, 60–84 years old, postmenopausal) according to age. The primary cells from alveolar bone were used for expression analysis after three passages and cultured in α‐MEM supplemented with 15% fetal bovine serum (FBS) and 1% penicillin/streptomycin at 37°C in a humidified atmosphere with 5% CO_2_. Once the BMSCs reached 80%–90% confluence, cells were cultured with fresh α‐MEM for 24 hr for further investigation. Next, the supernatant was collected and stored at −20°C (Nagasawa et al., 2002). To induce osteogenic differentiation, cells were cultured in an osteogenic medium containing complete medium supplemented with 50 μM ascorbic acid, 10 mM β‐glycerophosphate, and 10^−7^ M dexamethasone. Adipogenic induction was performed as follows: cells were cultured in complete medium containing 500 μM 3‐isobutyl‐1‐methylxanthine (IBMX), 10 mg/L insulin, 1 μM dexamethasone, and 0.2 mM indomethacin (Sigma, St. Louis, MO, USA). Both osteogenic and adipogenic differentiation media were replenished every 3 days.

### 4.3 Osteoclast formation

Bone marrow (BM) cells derived from femurs and tibias were harvested from 4‐week‐old SD rats. To remove adherent cells, BM cells were cultured with α‐MEM for 3 hr. Then, nonadherent cells in the supernatant were collected and seeded in 12‐well plates at a density of 1 × 10^6^ and cultured in complete medium with M‐CSF (20 ng/ml) for the first 3 days. Three days later, these cells were refreshed with supernatant supplemented with 15% FBS and 1% penicillin/streptomycin, M‐CSF (20 ng/ml) and RANKL (20 ng/ml) every 3 days (Nagasawa et al., 2002).

### 4.4 Trap staining and bone resorption

The detailed procedures were provided in the supplements.

### 4.5 Colony forming unit assay

The detailed procedures were provided in the supplements.

### 4.6 Alizarin red staining and Oil red O staining

The detailed procedures were provided in the supplements.

### 4.7 Cell senescence‐associated β‐galactosidase staining

The detailed procedures were provided in the supplements.

### 4.8 Immunofluorescence

The detailed procedures were provided in the supplements. For F‐actin ring staining, osteoclasts on creep plates were incubated with 1 ml PBS containing 1 μl Phalloidin‐TRITC Conjugate (AAT bioquest, AAT‐23102).

### 4.9 Transfection and dual‐luciferase reporter assay

293T cells were seeded in 24‐well plates (5 × 10^5^ in each well). After 24 hr, the cells were cotransfected with pGL3‐basic luciferase reporter vector (Promega, Madison, WI, USA) containing the 3′‐UTR fragment of E2F2 or RhoA, renilla vector (pRL‐TK; Promega, Madison, WI, USA), a scrambled miRNA control and miR‐31a‐5p mimics or miR‐31a‐5p inhibitors (GenePharma, Shanghai, China) by using Lipofectamine 2000 (Invitrogen). Luciferase activities were measured 48 hr after transfection by using the Dual‐Luciferase Reporter Assay System (Promega, Madison, WI, USA). Firefly luciferase activity was normalized to renilla luciferase activity for each sample.

### 4.10 Uptake of exosomes by osteoclasts

BMSCs were labeled with CM‐DiI cell‐labeling solution (Molecular Probes, Eugene, OR) according to the manufacturer's protocol. Briefly, BMSCs were harvested and resuspended in 1 ml of serum‐free α‐MEM. Next, 5 μl of DiI staining solution was added to the media and cells were incubated at 37°C for 20 min. The DiI cell‐labeling suspension was centrifuged at 251 g for 5 min and the supernatant was removed. Cells were washed two times with PBS and cultured in complete α‐MEM for 24 hr. Subsequently, the supernatant containing exosomes was harvested and incubated with osteoclasts at 37°C for 2 hr. Lastly, cells were fixed with 4% paraformaldehyde for 20 min and stained with DAPI for 1 min. Fluorescence images were captured under a fluorescence microscope (Leica Microsystems, Mannheim, Germany).

### 4.11 Isolation of exosomes derived from BMSCs

Young or elderly rat‐derived BMSCs were seeded on the dishes using the same cell numbers. Once adherent to the plate, BMSCs were washed with PBS twice and cultured in the same volume of serum‐free αMEM for 24 hr. Next, the supernatant was harvested and centrifuged at 300 *g* for 10 min, 2000 *g* for 15 min and 10,000 *g* for 30 min, discarding dead cells and collecting the supernatant each time. The supernatant was then centrifuged at 100,000 *g* for 70 min twice, removing the contaminating protein, and the pellet was collected each time and resuspended in PBS. Lastly, exosomes in the pellet were resuspended in 100 μl of PBS and stored at −80°C (Mead, & Tomarev, 2017).

### 4.12 Quantitative Real‐time PCR analysis

Total RNA was extracted from cells using Trizol reagent according to the manufacturer's instructions. Reverse transcription was performed with 1 μg of total RNA in a final volume of 20 μl using the PrimeScript RT reagent kit (Takara Bio, Shiga, Japan) according to the manufacturer's recommendations. miR‐31a‐5p of purified exosomes was extracted using the miRNeasy Serum/Plasma kit (QIAGEN, Valencia, CA, USA) according to the manufacturer's instructions. Expression of miR‐31a‐5p was detected by the All‐in‐One miRNA qRT‐PCR Detection Kit (GeneCopoeia, Rockville, MD, USA). The levels of each mRNA or miRNA were normalized to the β‐actin or U6 levels, respectively, while the exosomal miRNA levels were compared to the levels of spiked‐in ce‐miR‐39, which was applied as the reference using the miRNeasy Serum/Plasma Spike‐In Control kit (QIAGEN, Valencia, CA, USA). Each experiment was performed in triplicate. The primer sequences used in this study are listed in Table S1. The 2^−ΔΔCT^ method was used to quantify expression of the genes of interest.

### 4.13 Western blot

The detailed procedures were provided in the supplements.

### 4.14 Transfection of miR‐31a‐5p Inhibitors and Mimics

miR‐31a‐5p inhibitors, mimics, and the negative control (miR‐NC) were synthesized by GenePharma Co. Ltd. (Shanghai, China). For transient transfections, the miR‐31a‐5p inhibitors, mimics, and negative control (50 nM, final concentration) were transfected into BMSCs using Lipofectamine 2000 (Invitrogen, Carlsbad, CA, USA) in Opti‐MEM medium. In addition, BMSCs were added to the same volume of PBS as a control. Cells were harvested for further analyses at least 48 hr after transfection.

### 4.15 Histological observation

The detailed procedures were provided in the supplements.

### 4.16 Micro computed tomography (micro‐CT) analysis

The detailed procedures were provided in the supplements.

### 4.17 Statistical Analysis

The results are expressed as the means ± *SD*. Experiments were repeated independently at least three times. Statistical significance was assessed using Student's *t*‐test or ANOVA analysis. *p *<* *0.05 was considered statistically significant.

## CONFLICT OF INTEREST

The authors declare no conflict of interest.

## AUTHOR CONTRIBUTIONS

RYX performed experiments, analyzed data, and wrote the manuscript; XS, YMS, YF, and WWZ analyzed data and prepared the figures; TX, ZYF, PZ, and JC performed bioinformatics and statistical analyses; HBJ designed the experimental study, analyzed the data, and revised the manuscript. All authors read and approved the final manuscript.
