# Supplementary material for: MicroRNA‐31a‐5p from aging BMSCs links bone formation and resorption in the aged bone marrow microenvironment
Source: Aging Cell. 2018 Jun 12;17(4):e12794. doi: 10.1111/acel.12794 (PMC6052401; doi:10.1111/acel.12794)
Supplement: Supplementary file 1 [file ACEL-17-na-s001.doc]

**Supplementary Figure and Figure legends**

**
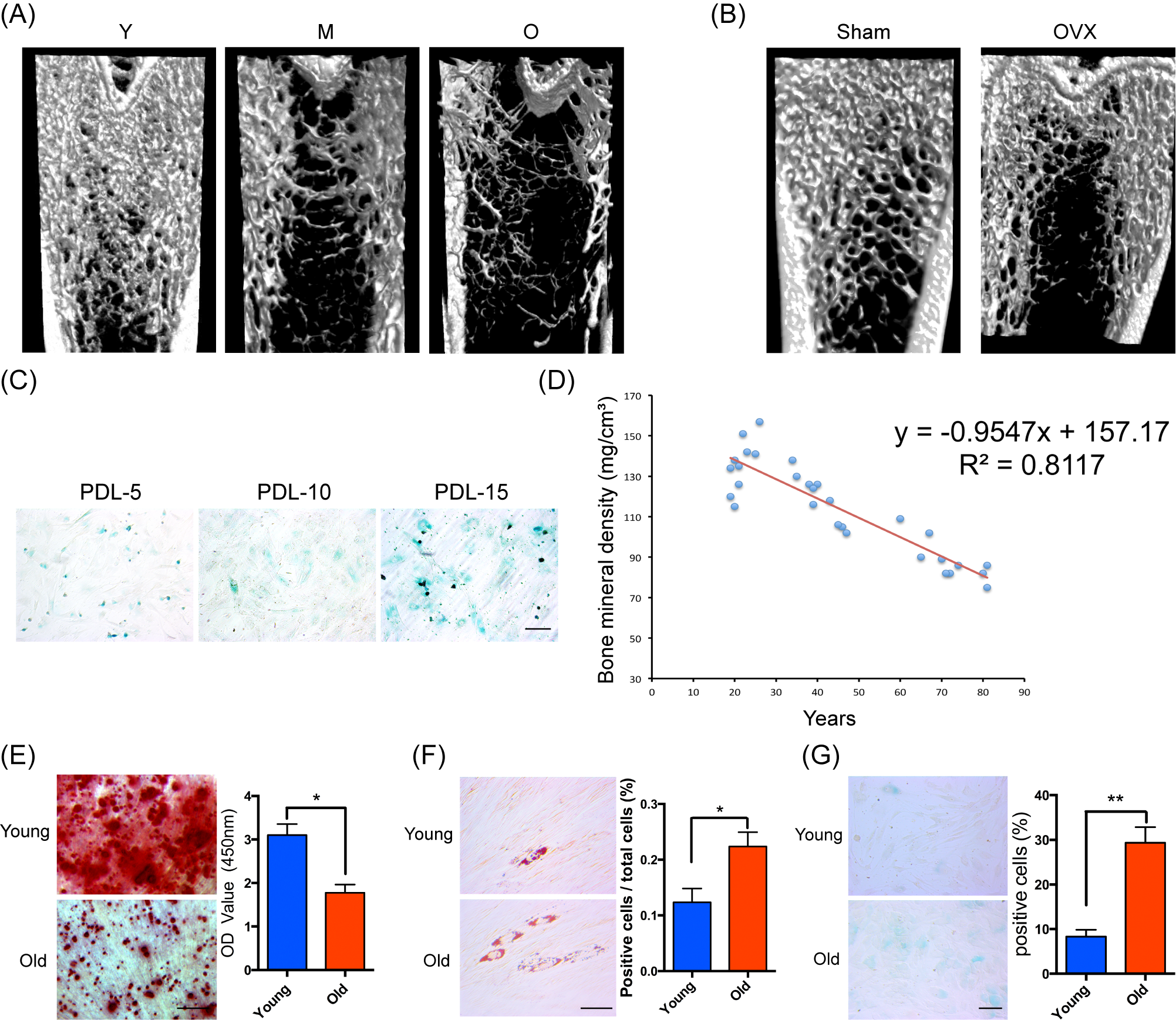
**

**Supplemental Figure 1.** Aged associated phenotype in different aging models. (A) Micro-CT revealed that the aged rat model and (B) estrogen-deficient aging model were successfully established. (C) SA-β-gal staining was performed at PDL-5, 10 and 15 points. (D) Correlogram showed inverse correlation between bone mineral density (BMD) and age. (E) Alizarin red staining, (F) oil red O staining, and (G) SA-β-gal staining were employed in young and aged human BMSCs. *^*^P*< 0.05, ^**^*P* < 0.01. Scale bars: 100 μm (C,E-G). Data are presented as the mean ± standard deviation, n = 3.


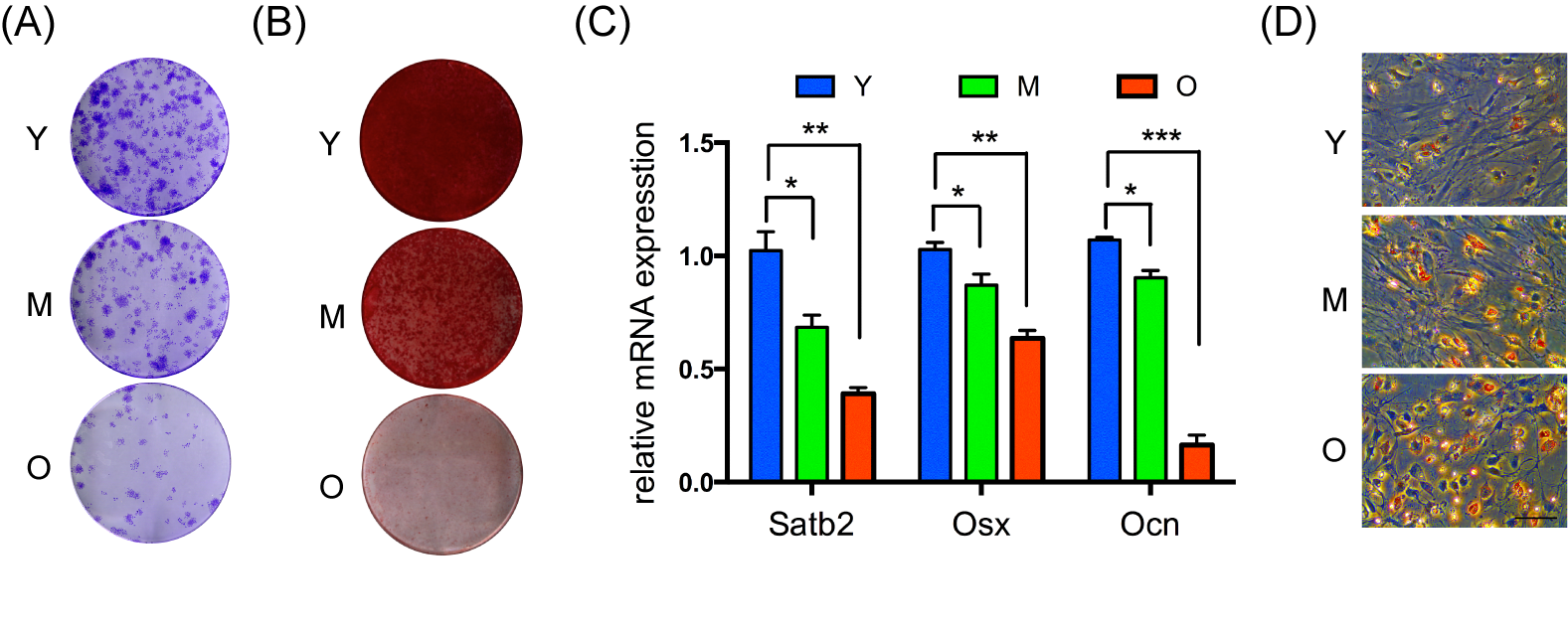


**Supplemental Figure 2.** Characteristics of BMSCs from aged rats. (A) Colony forming units were decreased in BMSCs from the O group compared to those from the Y and M groups. (B) Osteogenic differentiation of aged BMSCs was reduced by alizarin red staining. (C) qRT-PCR revealed that the SATB2 and osteogenic markers, Osx and Ocn, were significantly decreased in BMSCs from the O group. (D) Increased adipogenic ability of aged BMSCs was detected by oil red O staining. *^*^P*< 0.05, ^**^*P* < 0.01, ^***^*P* < 0.001. Scale bars: 100 μm (D). Data are presented as the mean ± standard deviation, n = 3.

**
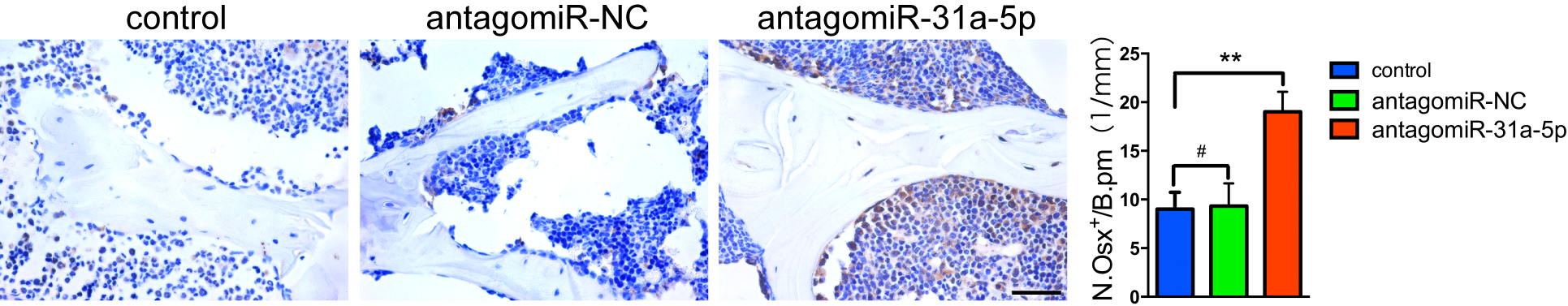
**

**Supplemental Figure 3.** Representative images of Osterix immunohistochemical staining and quantification of preosteoblast number on bone surface perimeter of control, antagomiR-NC and antagomiR-31a-5p group rats. Scale bar: 50 μm. N.Osx+/B.pm, number of osterix+ cells per bone surface. n=3 per group; *^#^P*> 0.05, ^**^*P* < 0.01.

**Supplemental Table**

Table S1. Sequence of primers for real-time RT-PCR

| Gene | Primer Sequence | Product size (bp) |
| --- | --- | --- |
| Satb2 | F:5-CACAGCTGCCATTTATGACGAGA-3  R:5-CTTCCAACGAAGCAGTTCACAGAG-3 | 124 |
| Osx | F: 5′-GGAGGCACAAAGAAGCCATA-3′  R: 5′-GGGAAAGGGTGGGTAGTCAT-3′ | 143 |
| Alp | F: 5’-TCATGTTCCTGGGAGATGGTATG-3’  R: 5’-GCATTAGCTGATAGGCGATGTCC-3’ | 124 |
| OPN | F: 5’- GCCGAGGTGATAGCTTGGCTTA-3’  R: 5’- TTGATAGCCTCATCGGACTCCTG -3’ | 136 |
| OCN | F: 5-TGCAAAGCCCAGCGACTCT-3  R: 5-TTGAGCTCACACACCTCCCTGT-3 | 159 |
| LPL | F: 5-ACAGTCTTGTAGCCCATGCT -3  R:5-AGCCAGTAATTCTATTGACCTTCTTGT-3 | 72 |
| PPARγ | F: 5-GAAGACATCCCGTTCACAAGA-3  R: 5-TGATGCTTTATCCCCACAGAC-3 | 207 |
| C/EBPα | F: 5-AAGAAGTGGGTGGATAAGAACAG-3  R: 5-GTTGCGCTGTTTGGCTTTATC-3 | 99 |
| β-actin | F:5-GGAGATTACTGCCCTGGCTCCTA-3  R:5-GACTCATCGTACTCCTGCTTGCTG-3 | 150 |

Table S2. Antibodies used

| Antibody | Dilution | Source | Code number |
| --- | --- | --- | --- |
| SATB2 | 1:1000 | Abcam | ab69995 |
| Nanog | 1:500 | Santa Cruz | SC-33760 |
| SOX2 | 1:500 | Santa Cruz | SC-17320 |
| OCT4 | 1:500 | Santa Cruz | SC-5279 |
| OSX | 1:1000 | Abcam | ab22552 |
| ALP | 1:1000 | Abcam | ab83259 |
| OPN | 1:800 | Bioworld | BS1264 |
| OCN | 1:800 | Bioworld | BS60987 |
| P16 | 1:500 | Abcam | ab51243 |
| P21 | 1:500 | Proteintech | 10355-1-AP |
| P53 | 1:800 | Proteintech | 10442-1-AP |
| RhoA | 1:800 | Cell Signaling Technology | 2117 |
| E2F2 | 1:1000 | Abcam | ab138515 |
| CD9 | 1:1000 | Abcam | ab92726 |
| CD63 | 1:1000 | Abcam | ab59479 |
| TSG101 | 1:1000 | Abcam | ab30871 |
| Calnexin | 1:800 | Proteintech | 10427-2-AP |
| β-actin | 1:400 | Boisynthesis Biotechnology | Bs-oo61R |

**Experimental procedures**

**Colony forming unit (CFU) assay**

A total of 200 nucleated cells were seeded in six-well plates in triplicate. The cells were cultured in complete medium for 10 days. Cell colonies were fixed in 4% paraformaldehyde and stained with a crystal violet solution (Sigma, St. Louis, Mo). Aggregates of 50 or more cells were counted as colonies.

**Alizarin red staining and Oil red O staining**

Following osteogenic induction for 10 days *in vitro*, BMSCs were stained using alizarin red at room temperature for 10 minutes and then rinsed with PBS. The deposition of calcium was identified under a light microscope. Calcified nodules were eluted with 10% cetylpyridinium chloride (CPC), and the absorbance was calculated at 562 nm and quantified after controlling to cell numbers of each group. Differentiated adipocytes from BMSCs were induced for 14 days and stained with Oil red O for 1 h. The proportion of positively stained cells in the total area was measured using ImageJ software. Five random fields from each sample were captured under a light microscope and chosen for quantification.

**Cell senescence-associated β-galactosidase staining**

Cellular senescence-associated β-galactosidase activity (SA-β-gal) was performed according to the instructions on the β-gal staining kit (GenMed Scientifics, Inc., Shanghai, China). Briefly, BMSCs were seeded in 12-well plates at a density of 5×10^4^ cells/cm^2^ overnight. Next, cells were washed with PBS and incubated in staining solution for 16 h at 37°C. Five randomly selected fields were chosen for quantification of SA β-gal positive cells using inverted microscopy.

**Immunofluorescence**

After grown on coverslips BMSCs were cultured for 24 h at 37°C and then fixed with 4% paraformaldehyde, permeabilized in PBS containing 1% Triton X-100 for 30 min at room temperature and pre-incubated with goat serum to block nonspecific staining. Following overnight incubation with primary antibodies at 4°C, cells were washed with TBS 10 min for three times and incubated with Cy3-labeled secondary IgG at 37◦C for 1 h. Nuclei were labeled with 4′, 6-diamidino-2-phenylindole (DAPI) at room temperature for 1.5 min. Fluorescence images were captured under fluorescence microscope (Leica Microsystems, Mannheim, Germany).

**Trap staining and bone resorption**

Cell tartrate-resistant acid phosphatase (TRAP) staining was employed according to the manufacturer’s instructions for the TRAP-kit (Sigma-Aldrich, St. Louis, MO, USA). Cells were fixed with 4% paraformaldehyde and incubated with staining solution for 10 minutes. Mature osteoclasts were counterstained with methylene blue and identified as more than two positively stained nuclei. Non-adherent cells were cultured on a dentin disk, which was placed in the 12-well plates. After 12 days, the resorption pits of the bone slices were visualized with the Leica Application Suite (Leica, Mannheim, Germany).

**Western blot**

Western blot was performed according to previous methods (*Fu et a*l. 2014). Cells were lysed with RIPA buffer (Beyotime, Shanghai, China) containing 10 mM protease inhibitor (PMSF; Beyotime). Protein lysate was loaded onto 10–15% SDS-PAGE gels and then transferred to PVDF membranes (Millipore, Billerica, MA, USA). Membranes were blocked using 5% fat-free milk for 2 h and subsequently incubated with primary antibodies overnight. Detailed information regarding the primary antibodies is listed in Table S2. After washing with TBST three times, the membranes were incubated for 1 h with the corresponding horseradish peroxidase-conjugated secondary antibodies (1:10,000). The blots were visualized using an ECL detection kit (Millipore). Quantitative analysis of the western blotting results was carried out using ImageJ software. In each group, the relative protein levels were quantified as the ratio of the level of the protein of interest to the level of β-actin.

**Micro computed tomography (micro-CT) analysis**

The micro-architectural properties of the distal femur were analysed using the micro-CT system (Skyscan 1176, Kontich, Belgium). Bones were scanned at a high resolution (18 μm) with an energy of 50 kV and 456 μA. We applied NRecon v1.6 and CTAn v1.13.8.1 software to reconstruct and analyse the 3D images of the bone. The region of interest (ROI) was defined as the cancellous bone of the distal femur. To evaluate the bone structure, the following four parameters were calculated: the bone volume ratio (BV/TV, %), trabecular thickness (Tb.Th.), trabecular number (Tb.N.) and trabecular separation (Tb.Sp.). For cortical bone, we selected the region of mid-diaphysis on the 10% femoral length to analyse the cortical thickness (Ct. Th). For bone mineral density measurement, we selected bone area under the first mandibular molar to analyse cancellous bone.

**Histological observation**

Bone specimens were fixed with 4% paraformaldehyde for at least 24 h. Subsequently, bone samples were decalcified in 10% ethylene diaminetetraacetic acid (EDTA), embedded in paraffin wax and sectioned into 4-μm-thick slices. The bone structure of the distal femur was observed by HE and masson’s trichrome staining. The quantification of osteoblast numbers is presented as graphs of osteoblasts per millimeter of bone perimeter. TRAP staining was performed according to the instructions of the TRAP-kit (Sigma-Aldrich, St. Louis, MO, USA), and the positive cells were counterstained with methylene blue. For the quantification of TRAP staining, data are presented as graphs of quantification of OC.N/B.Pm (osteoclast number per bone perimeter) and OC.N/BS (osteoclast number per bone surface). For immunohistochemical staining, the slides were cultured with 3% H2O2 for 20 mins to eliminate endogenous peroxidase. Then, the slides were washed with PBS three times and incubated with normal goat serum for 40 mins at 37 °C. Polyclonal rabbit anti-rat OSX antibodies (1:300, ab22552, Abcam) were used as primary antibodies and added on tissue slides at 4 °C overnight. Subsequently, the slides were treated with secondary antibodies and diaminobenzidine (DAB) to detect the immunoactivity, and the slides counterstained with hematoxylin.
